# Draft Genome Sequence of Triclosan-Resistant Cystic Fibrosis Isolate *Achromobacter xylosoxidans* CF304

**DOI:** 10.1128/genomeA.00865-15

**Published:** 2015-07-30

**Authors:** Julie Jeukens, Luca Freschi, Irena Kukavica-Ibrulj, Dao Nguyen, Roger C. Levesque

**Affiliations:** a Institut de Biologie Intégrative et des Systèmes (IBIS), Université Laval, Québec, Québec, Canada; b Department of Medicine, McGill University, Montreal, Québec, Canada

## Abstract

*Achromobacter xylosoxidans* is an emerging opportunistic pathogen. Here, we present the genome sequence of cystic fibrosis isolate CF304. Assembly resulted in 29 contigs adding up to 6.3 Mbp. This is the second genome sequence for a cystic fibrosis isolate, and little is known about the genetic basis of pathogenicity in this organism.

## GENOME ANNOUNCEMENT

*Achromobacter xylosoxidans* is an environmental bacterium, often considered an emerging opportunistic pathogen in the context of cystic fibrosis (CF) lung infections (1, 2), with a prevalence estimated at 10 to 20% among CF patients (3, 4). Similar to *Pseudomonas aeruginosa*, the most common cause of lung infection among CF patients, it can establish persistent chronic infections associated with inflammation (4, 5), produce biofilms, and readily acquire antibiotic resistance (3). Here, we present the genome sequence of *A. xylosoxidans* CF304, which was the dominant isolate in the sputum sample from an adult CF patient. Its ability to grow on *Pseudomonas* isolation agar showed that it was resistant to irgasan, or triclosan, a broad-spectrum antimicrobial agent. This compound, which can induce multidrug resistance *in vitro* in *P. aeruginosa* (6), is widely used for disinfection purposes and may promote multidrug resistance in the environment (7).

*A. xylosoxidans* CF304 was isolated on Difco *Pseudomonas* isolation agar (BD, Sparks, MD). Genomic DNA was isolated from an overnight culture using the DNeasy blood and tissue kit (Qiagen, Hilden, Germany). Genomic DNA (500 ng) was mechanically fragmented for 40 s using a Covaris M220 (Covaris, Woburn, MA, USA) with default settings. Fragmented DNA was transferred to a PCR tube, and library synthesis was performed with the Kapa Hyper prep kit (Kapa Biosystems, Wilmington, MA, USA), according to the manufacturer’s instructions. TruSeq HT adapters (Illumina, San Diego CA, USA) were used to bar code the library, which was sequenced in 1/48 of an Illumina MiSeq 300-bp paired-end run at the Plateforme d’ Analyses Génomiques of the Institut de Biologie Intégrative et des Systèmes (Laval University, Quebec, Canada). The complete procedure was done a second time, using the original isolate. The two sequencing data sets produced were combined and assembled *de novo* with the A5 pipeline (8).

The CF304 genome assembly consists of 29 contigs (median coverage, 61×), for an estimated total size of 6,302,145 bp. Core genome phylogeny of all 6 available *A. xylosoxidans* genomes with the Harvest suite (9) showed that this novel genome sequence is closely related to that of the other *A. xylosoxidans* clinical strains. Accessory genome analysis with Roary (10) located 5 gene families that are not present in other *A. xylosoxidans* genomes: 4 are hypothetical proteins, and 1 is an EF-Tu elongation factor. In *P. aeruginosa*, protein FabV (locus NP251640, or PA2950) was shown to be responsible for triclosan resistance (11). Using a BLASTn search (12), we found one homologous sequence for this protein in the CF304 genome (81% identity). The same result was found in all available *A. xylosoxidans* genomes.

This is the second published genome from an *Achromobacter* CF isolate (2). As the importance of this pathogen is increasingly recognized in CF and other opportunistic infections, this new genome represents a resource for further studies on the genetic basis of pathogenicity in this organism.

### Nucleotide sequence accession numbers.

This whole-genome shotgun project has been deposited at DDBJ/EMBL/GenBank under the accession no. LFHA00000000. The version described in this paper is version LFHA01000000.
